# Training simulated patients: evaluation of a training approach using self-assessment and peer/tutor feedback to improve performance

**DOI:** 10.1186/1472-6920-9-37

**Published:** 2009-06-29

**Authors:** Jennifer Perera, Joachim Perera, Juriah Abdullah, Nagarajah Lee

**Affiliations:** 1 Department of Pathology and Chairperson of Medical Education Research group on student learning, International Medical University (IMU), Kuala Lumpur, Malaysia; 2 Department of Human Biology and chairman of the Medical Education Research group on assessments, IMU, Kuala Lumpur, Malaysia; 3 Department of Clinical Sciences, Coordinator Clinical Skills Unit, IMU, Kuala Lumpur, Malaysia; 4 Department of Community Medicine, IMU, Kuala Lumpur, Malaysia

## Abstract

**Background:**

Most medical schools use simulated patients (SPs) for teaching. In this context the authenticity of role play and quality of feedback provided by SPs is of paramount importance. The available literature on SP training mostly addresses instructor led training where the SPs are given direction on their roles. This study focuses on the use of peer and self evaluation as a tool to train SPs.

**Methods:**

SPs at the medical school participated in a staff development and training programme which included a) self-assessment of their performance while observing video-tapes of their role play using a structured guide and b) peer group assessment of their performance under tutor guidance. The pre and post training performance in relation to authenticity of role play and quality of feedback was blindly assessed by students and tutors using a validated instrument and the scores were compared. A focus group discussion and a questionnaire assessed acceptability of the training programme by the SPs.

**Results:**

The post-training performance assessment scores were significantly higher (p < 0.05) than the pre-training scores. The degree of improvement in the quality of feedback provided to students was more when compared to the improvement of role play. The acceptability of the training by the SPs was very satisfactory scoring an average of 7.6 out of 10. The majority of the SPs requested the new method of training to be included in their current training programme as a regular feature.

**Conclusion:**

Use of structured self-reflective and peer-interactive, practice based methods of SP training is recommended to improve SP performance. More studies on these methods of training may further refine SP training and lead to improvement of SP performance which in turn may positively impact medical education.

## Background

The use of simulated patients (SPs) in medical education has been described as early as 1968 and a number of advantages have been identified, over the use of real patients [1]. Currently more than 80% of medical schools use SPs for training and assessing the competency of health professionals [2]. The advantages in using SPs in training and assessments of medical students are presented in a detailed overview by Barrows [3]. In the educational setting, when SPs are used for training of health professionals, they offer students the opportunity to learn and practice communication skills, history taking and examination techniques in a supportive, low risk (in relation to the well being of patients) and authentic environment. Trained SPs, in addition, are able to provide feedback on students' skills on building rapport with patients, manner of speech and language, interview structure and style etc.

When SPs are used for role playing clinical scenarios during examinations, the emphasis is on standardization of the SPs to ensure consistent role play as this is important for creating fair and equal circumstances for examinees. This is particularly relevant during high-stakes examinations. However, when viewed in the context of medical education, *authenticity *of role play and ability to provide the students with useful *feedback *are important, in terms of the quality of learning during SP contact learning sessions. Therefore, training of SPs in these areas is of paramount importance prior to using them in medical education.

A variety of methods for training SPs have been described such as demonstrations and video-clips on role play for orientation on SP technique, observation of real patients being interviewed and examined by physicians, coaching by experienced SPs or professional actors/actresses, and feedback by students and teaching faculty on SP performance [1]. A recently published concise text further provides detailed instructions on the training of SPs for teaching and assessments [4]

### Current status of SP training at the International Medical University

The International Medical University (IMU) has an integrated system-based curriculum for medical undergraduates in the pre-clinical phase which runs over five semesters (two and a half years). During this phase of the curriculum, SPs are used in both teaching and assessments and the use of real patients is minimal. The SP related teaching and learning activities are conducted with small groups of 5–6 students. The SPs enact or role play clinical scenarios relevant to different organ systems with individual students and in addition provide feedback on their performance during the history taking and physical examination learning sessions. The feedback provided by the SPs mainly focus on communication skills and patient etiquette. The tutors provide feedback on specific content knowledge and clinical skills during these supervised learning sessions, although not all learning sessions are supervised by tutors.

Currently IMU has a pool of 70 SPs and they undergo conventional small group training using video clips and group practice sessions etc. following recruitment, prior to participating in teaching. Case scenarios and videotapes are used for training over a day long workshop, delivered once a year. Experienced SPs, enact case scenarios during these demonstration sessions in the training programme. In addition, at the beginning of each organ system module in the curriculum, the SPs receive a short briefing of 30 minutes on the cases that they are expected to role play during the clinical skills teaching programme related to each system. Although SPs have been used for over 10 years in clinical skills teaching at IMU, the performance of these SPs has not been reviewed on a regular basis or in a structured manner either by students, tutors or curriculum managers. Thus it was important to measure and improve their current performance. In view of this lack of information, an evaluation of SP performance at teaching sessions was conducted. This identified some deficiencies in role play of the SPs and effectiveness of providing feedback to the students. Based on this information, retraining of SPs using an innovative approach based on self and peer assessment and feedback was designed.

## Methods

### Study population

Nine simulated patients (3 men and 6 women), who regularly participate in teaching, were selected for this pilot training study. They were scheduled to participate in the haematology system clinical skills teaching sessions in the third semester of the medical undergraduate curriculum. The ages of the SPs ranged from 28 to 67 years and their duration of work experience as SPs ranged from 2–5 yrs. The background of the SPs varied widely and included housewives, retired health care workers, secretaries, and an ex-engineer. The SPs were informed of the pilot study and their consent for participation was obtained.

### Study design

The three sequential components of the study, namely initial review of SP performance, training and post training performance review were conducted in a stepwise manner as follows (summarised in Table 1)

**Table 1:** Study design

Step 1	Videotaping 8 consultations of each SP in haematology system
Step 2	Review of videotapes by students and tutors using modified MaSP instrument

Step 3	Self assessment by SPs using their recorded videotapes

Step 4	Tutor/peer feedback session with SPs using videotapes

Step 5	Videotaping 8 consultations of each SP in the Gastrointestinal system

Step 6	Re-review of videotapes by students and tutors using modified MaSP instrument

Step 7	Comparing scores of SP obtained pre and post training

#### Step 1

During the haematology system history taking sessions, eight consultations between medical students and SPs were video-taped per SP. Each SP enacted a different clinical scenario and all students rotated between the SPs.

#### Step 2

The video tapes were reviewed using the modified MaSP instrument validated by the Medical Education Unit of the Maastricht University [5]. The students and tutors who reviewed the tapes were blinded to the experiment. The instrument was modified and revalidated to suit the institutional setting after an initial study [6]. The modifications, primarily, were in terms of simplifying the language and removing items which were not relevant to the institution. The instrument consisted of two main parts. The first part contained ten items (No.1–10) and assessed the authenticity of role play and SP behaviour. The second part had a further ten items (no 11–20) and assessed the quality of feedback provided to the student doctor at the end of the consultation. Item 21 was used for providing an overall score out of 10 for the entire consultation (see Additional file 1).

The performance of each SP was reviewed by 60 students, as a minimum of 30 reviewers has been previously recommended for maintaining reliability and validity [5]. Prior to review, the items in the instrument were explained to the student reviewers. Each reviewer (students and tutors) evaluated a single 7 minute interview session of three different SPs (a total of 3 interview sessions) during a time allocated by the clinical skills unit, where video viewing facilities were arranged for small groups (10 students/per group). The students evaluated the performance of each SP independently under the supervision of one of the authors.

#### Step 3

The SPs were provided with a self assessment form designed using the MaSP instrument and reviewed their performance while viewing videotaped recordings of their interviews. SPs were provided with as much time as they needed for self review. All the SPs spent over one hour on their self review as each videotape contained eight, 7 minute haematology interview sessions, in which they enacted the same case scenario with eight different students.

#### Step 4

The SPs were invited to a tutor and peer feedback meeting where the recorded interviews were viewed. All nine participated. One 7 minute history taking session per SP was used for this feedback meeting. During the session the SPs commented on the performance of their peers, followed by tutor feedback.

#### Step 5

Steps 1 and 2 were repeated 2 months after the training programme, during the gastrointestinal system history taking sessions, and the same students and tutors reviewed the SP performance using the MaSP instrument. The reviewers were blinded to the training experiment to avoid bias during re-assessment.

At the end of the pilot study, a focus group discussion was conducted and an anonymous questionnaire (see Additional file 2) was used to explore the SPs' perceptions on the usefulness and acceptability of the new training programme.

The effectiveness of the self and peer assessment method of training was assessed objectively by comparing the scores that individual SPs received during pre-training and post-training clinical skills learning sessions.

The modified MaSP questionnaire had a Cronbach's alpha coefficient for the various items which was greater than 0.650 indicating acceptable reliability after modification. The convergent validity using the correlation analysis between the individual items and the overall assessment score as proposed by Narver & Slater [7] provided values ranging from 0.318 to 0.612 which indicated good convergent validity. The concurrent validity using the independent sample t-test, comparing the differences in '*individual item scores' *and '*overall evaluation score*' showed that there were significant differences between the two groups (favorable overall assessment and unfavorable overall assessment). This is evidence for good concurrent validity. The goodness of fit of the instrument based on the confirmatory factor analysis was as follows: Goodness of Fit Index (GFI) = 0.88, Adjusted Goodness of Fit Index (AGFI) = 0.87, Root Means Square Error of Approximation (RMSEA) = 0.061, Normed Fit Index (NFI) = 0.88, Critical Fit Index (CFI) = 0.89, Parsimonious Normed Fit Index (PNFI) = 0.77, and Parsimonious Goodness if Fit Index (PGFI) = 0.84.

Approval for the study was granted by the Research and Ethics Committee of the International Medical University. Informed verbal consent was obtained from the simulated patients and reviewers (students and tutors) who participated in the study.

## Results

### Effect of role play and feedback on SP performance

Table 2 provides data on average scores received for the authenticity of role play and quality of feedback during pre and post training evaluations and the *p *value derived from the paired *t *test. The majority of the SPs showed significant improvement after the training (p < 0.05) in both role play and feedback provision as indicated by reviewers' scores. When individual SPs are considered the improvement in the quality of feedback provided to students was more when compared to that of role play. The pre and post training overall average scores (item 21 of the instrument) for the SP consultations were 7.06 (SD = 0.92) and 8.26 (SD = 0.99) respectively showing a significant improvement (p < .0.027) when analysed using the paired *t *test.

**Table 2:** Pre and post training assessment scores of reviewers for authenticity of role play and quality of feedback of SPs, when assessed by modified MaSP instrument

**SP No**	**Authenticity of role play**			**Quality of feedback**		
	**Pre-training scores ± SD**	**Post-training scores ± SD**	***P *value**	**Pre-training scores ± SD**	**Post-training scores ± SD**	***p *value**

1	2.56 (0.30)	3.01 (0.35)	0.01	2.93 (0.32)	3.10 (0.39)	0.09
2	3.00 (0.31)	3.13(0.37)	0.07	2.78 (0.33)	3.09 (0.37)	0.03
3	3.07 (0.29)	3.42 (0.33)	0.04	2.79 (0.36)	3.11 (0.42)	0.02
4	2.98 (0.34)	3.47 (0.35)	0.01	2.93 (0.39)	3.19 (0.43)	0.01
5	3.37 (0.41)	3.42 (0.38)	0.10	3.04 (0.27)	3.21 (0.29)	0.02
6	3.15 (0.39)	3.67(0.36)	0.008	3.01 (0.36)	3.18 (0.42)	0.04
7	3.15 (0.41)	3.37 (0.46)	0.07	3.04 (0.41)	3.21 (0.40)	0.04
8	3.49 (0.37)	3.77 (0.42)	0.05	3.19 (0.36)	3.52 (0.39)	0.03
9	3.11 (0.29)	3.65 (0.47)	0.01	2.81 (0.29)	3.27 (0.39)	0.001

**Average**	**3.11 (0.35)**	**3.46(0.39)**	**0.043**	**2.97 (0.31)**	**3.19 (0.29)**	**0.047**

### Perceptions and acceptability

Results of the focus group discussion showed that the SPs were satisfied with the method of training. The questionnaire analysis showed that the average overall score received for the training programme was 7.6 out of 10. Two SPs among the nine felt embarrassed when others were viewing their performance, but none felt harassed or uncomfortable during this peer review. Seven of the nine SPs reported that they learnt new areas for improving performance during self evaluation and the peer/tutor feedback session. The SPs perceived that the degree of learning was more during peer/tutor feedback session than during their individual self assessment.

Six SPs provided free text comments; four requested similar training programmes either regularly or once every six months. "Improved confidence", "identified weaknesses particularly in relation to feedback", "motivated to perform better" "prior to training it was quite a blur" were other positive statements found among the comments. There were no negative comments on the method of training. The following interesting remarks were also present among the free text comments; "simply learned by observing performance of others", "the presence of tutor was important during group viewing to clarify issues", small group learning was useful".

## Discussion

It has been shown that direct involvement of learners in assessing their work is highly effective in enhancing learning [8]. The usefulness of self evaluation in improving learning has been conceptualized by Butler and Winnie [9]. Furthermore, practice based feedback has been found to be useful for improved learning [10]. More importantly, it has been reported that self evaluation and feedback are interdependent. One of the key papers that recognized this interdependence of self evaluation and feedback during learning has been published by Sadler [11] where three conditions have been identified as essential for learners to benefit from feedback. The learners must, a) possess a concept of the standard or reference level being aimed for, b) compare their current level of performance with that of the standard and c) engage in appropriate action which leads to closure of this gap. These principles were used in designing the new SP training programme.

Several factors may have contributed to the success of this training programme. Kaufman in his paper on learning theory summarizes the steps in adult learning or "andragogy" [12] as follows; for effective learning to take place the adult learner (SP in the current study) has to, actively engage with their existing self knowledge (doing the role play in student learning sessions), involve in diagnosing their own needs (structured self assessment during individual videotape viewing), identify strategies to use external resources, and reconstruct learning and internalize the outcomes (during tutor and peer feedback session). Kauffman further stated that, for learning to be effective, the learners should be provided with a supportive, practice based environment. In the current SP training programme this was provided through viewing of recorded interviews in small groups with the availability of tutor support (step 4). In addition, the development of self direction and regulation can be facilitated by structured learning which makes goals explicit [13]. The structured self reflection guide used during self assessment assisted SPs in identifying required standards (step 3). Studies have shown that self-assessment alone may not be an accurate measure of performance due to several reasons [14], namely misapprehension (where learners are not clear about expectations), and self deception due to being over confident. Therefore, learning during self assessment needs to be strengthened by performance based feedback [15]. Thus, the current method of training of SPs has addressed the main requirements for effective practice based learning.

### Learner acceptability

An evaluation of a new training method is incomplete without an inquiry into acceptability by the focused participants. In view of the limited experience of SPs on self assessment and feedback in a formal setting, and when viewed in the context of their varied social and professional background, the training method could be perceived as a challenge. However, the SPs recognised the importance of such training and desired similar training as a regular biannual feature in their programme of learning.

## Conclusion

The inclusion of guided self assessment and reflection was found to be useful in SP training. In addition peer and tutor feedback appeared to be non-threatening to the SPs, when completed in a supportive small group setting. When feedback and self reflection occurred in a practice based setting the learning appeared to be significantly higher. All these factors may have contributed to the success of the SP training programme as suggested by significantly higher post training scores received by SPs, when compared to pre training scores during evaluation. Therefore the use of self-reflective, peer-interactive, practice based SP training can enhance the quality of SP performance, when completed as part of an ongoing professional development programme. Further evaluation of this approach with a larger number of SPs from different educational and professional experiences may further refine SP training and lead to improvement of SP performance which in turn is expected to positively impact medical education.

### Limitations of the study

The "Hawthorne effect" created by the extra attention provided to the group of SPs may have contributed to their improved performance. A few students reviewed their own encounters and this may have led to some bias although this was minimised by having more than the recommended 30 students to review a single SP performance. In addition the study is limited by the small number of SPs studied and therefore results cannot be generalized unless the experiment is conducted on a larger population of SPs. The validity of the study could have been improved with the inclusion of a control group of SPs.

## Competing interests

The authors declare that they have no competing interests.

## Authors' contributions

JeP conceived the idea for the study, designed, collected data and drafted and revised the manuscript, JoP collected and interpreted data and reviewed all versions of the manuscript, JA reviewed the study design, collected data and reviewed the draft manuscript and NL assisted in designing the study and did the statistical analysis of data. All authors approved the final version of the manuscript

## Pre-publication history

The pre-publication history for this paper can be accessed here:



## Supplementary Material

Additional file 1 **Modified MaSP instrument**. The modified MaSP instrument was used by reviewers to evaluate SP performance pre and post training. Click here for file

Additional file 2 **Questionnaire used for determining SP perceptions on training method**. Instrument used for evaluation of SP perceptions on the training programme Click here for file
