# Supplementary material for: Training simulated patients: evaluation of a training approach using self-assessment and peer/tutor feedback to improve performance
Source: BMC Med Educ. 2009 Jun 29;9:37. doi: 10.1186/1472-6920-9-37 (PMC2711071; doi:10.1186/1472-6920-9-37)
Supplement: Additional file 1 — Modified MaSP instrument. The modified MaSP instrument was used by reviewers to evaluate SP performance pre and post training. [file 1472-6920-9-37-S1.doc]

**Appendix 1:**

**Modified MaSP instrument used by reviewers to evaluate SP performance**

| **The assessment of simulated patients(SP) by tutors/students using the validated and modified MaSP instrument**  **SP role name: Module** :  **Tape was assessed by** : (tick as appropriate)  Student doctor: Fellow students observers: Tutor: | | | |
| --- | --- | --- | --- |
|  | | | |
| **Note**: This survey gives you an opportunity to anonymously express your assessment or evaluation of your experience regarding the SP and SP contact session.  Please answer the questions objectively using the scale given and with written comments where applicable. | | **Rating Scheme:**  CD -Complete disagreement MD - Moderate disagreement  MA - Moderate agreement  CA - Complete agreement | |
|  |  | |  |
| **A.** | ***Authenticity: during the consultation*** | |  |
| 1 | SP appears authentic(real) | | CD MD MA CA |
| 2 | SP overplayed/ underplayed so that the student knew that SP was clearly role playing | | CD MD MA CA |
| 3 | SP appears to withhold information unnecessarily | | CD MD MA CA |
| 4 | SP stays in her/his role all the time and did not forget the role | | CD MD MA CA |
| 5 | SP tries to test /challenge the student | | CD MD MA CA |
| 6 | SP answers questions in a natural manner | | CD MD MA CA |
| 7 | SP’s appearance fits the role | | CD MD MA CA |
| 8 | SP’s reaction shows whether he/she listens to the student | | CD MD MA CA |
| 9 | SP drifted or does not pay full attention when the student is asking questions | | CD MD MA CA |
| 10 | SP starts conversation with students during time out | | YES NO |
| **B.** | ***Feedback after the consultation*** | |  |
| 11 | SP communicated to students how she/he felt during the consultation (eg. greeting, eye contact, listening skills etc) | | CD MD MA CA |
| 12 | SP communicates how he/she felt about their smooth or mechanical interview style | | CD MD MA CA |
| 13 | SP stimulates the student to ask questions during the feedback | | CD MD MA CA |
| 14 | SP gives feedback on medical jargon/ complicated terms used | | CD MD MA CA |
| 15 | SP gives feedback from the particular consultation | | CD MD MA CA |
| 16 | SP speaks about his/her role in the first person using “I” | | CD MD MA CA |
| 17 | SP gives constructive criticism | | CD MD MA CA |
| 18 | SP compares student with other students | | CD MD MA CA |
| 19 | SP gives feedback about knowledge aspects on medical issues | | CD MD MA CA |
| 20 | SP is friendly and not professional to the student | | CD MD MA CA |
| 21 | What mark (out of 10) would you give the SP for this simulated SP contact | | ……from (1-10) |
