# Supplementary material for: Training simulated patients: evaluation of a training approach using self-assessment and peer/tutor feedback to improve performance
Source: BMC Med Educ. 2009 Jun 29;9:37. doi: 10.1186/1472-6920-9-37 (PMC2711071; doi:10.1186/1472-6920-9-37)
Supplement: Additional file 2 — Questionnaire used for determining SP perceptions on training method. Instrument used for evaluation of SP perceptions on the training programme [file 1472-6920-9-37-S2.doc]

**Appendix 2:**

**Questionnaire used for determining SP perceptions on training method**

**Instrument used for evaluation of SP perceptions on the training programme**

Dear SPs, This questionnaire is to obtain your views for improving teaching of clinical skills which are important in the practice of medicine. Recently we introduced open self assessment and peer assessment in training SP. We want your honest opinion on the process, benefits and negative aspects.

**Rating scheme:**

1. - Always
2. - Frequently
3. - Sometimes
4. - Occasionally
5. - Never
6. My experience in school or university or college

| 1 | I have assessed my work/performance in **private** in a formal manner previously in pre-university education | 1 2 3 4 5 |
| --- | --- | --- |
| 2 | I have assessed my colleagues’ work in **private** in a formal manner in pre-university education | 1 2 3 4 5 |
| 3 | I have self assessed my work performance **openly** in front of my peers (class) during pre-university education | 1 2 3 4 5 |
| 4 | I have self assessed my colleagues’ work performance **openly** in front of peers (class) during pre-university | 1 2 3 4 5 |

1. SP training workshop : my experience

| 5 | I felt shy when providing feedback on myself to the group | 1 2 3 4 5 |
| --- | --- | --- |
| 6 | I learnt many things that I did wrong when I did self evaluation | 1 2 3 4 5 |
| 7 | I felt awful when I was providing feed back to others on their performance | 1 2 3 4 5 |
| 8 | I learnt many things when my peers/doctors evaluated me which I would never have thought of myself | 1 2 3 4 5 |
| 9 | I felt uncomfortable when others were providing feedback on my performance | 1 2 3 4 5 |
| 10 | I felt harassed when others were providing feedback on my performance | 1 2 3 4 5 |
| 11 | I used the points shown during self and peer evaluation to improve my performance at practice CSU session | 1 2 3 4 5 |
| 12 | Any specific aspect that I was able to improve on when the self evaluation and peer evaluation was done on role play and feedback to students  (You may select more than one by ticking √)   1. **On giving feedback to students**    - Empathy    - Eye contact    - Clarity of questions    - Facial expressions    - Paraphrasing    - Medical jargon    - Addressing concerns    - Closing interview    - Listening    - Interview style    - Addressing concerns | **2)On role play**   - Authenticity of role - Withholding information - Forgetting the role - Challenging the student - Drifting away from the role |

***C. My rating of the training workshop on a scale of 1 to 10 is*** …….……

***D*** ***Any additional comments of your own experience in peer and self evaluation during the workshop for learning SP skills***

………………………………………………………………………………………………………………………………………………………………………………………………………………………………………………………………………………………………………………………………………………………………………………………………………………………………………………………………………………………………………………………………………………………………………………………………………………………………………………………………………………………………………………………………………

**Thank you for sharing your views and completing the questionnaire**
